# Indications and clinical outcomes in patients with soft-tissue sarcoma attached to the bone receiving tumorendoprosthetic replacement after tumor resection

**DOI:** 10.1007/s00402-024-05735-2

**Published:** 2025-03-10

**Authors:** Nina Myline Engel, Arne Streitbürger, Jendrik Hardes, Wiebke K. Guder, Lars Erik Podleska, Markus Nottrott, Recep Öztürk

**Affiliations:** Department of Orthopedic Oncology, Universitätsklinikum Essen (AöR), Hufelandstraße 55, 45147 Essen, Germany

**Keywords:** Soft-tissue sarcoma, Bone invasion, Bone resection, Endoprosthesis replacement, Limb salvage

## Abstract

**Introduction:**

Bone resection followed by endoprosthetic reconstruction (EPR) in the treatment of soft tissue sarcoma (STS) is rare and associated with unique challenges. This study aimed to analyze the indications, results and factors affecting the results of these cases.

**Materials and methods:**

Twelve patients (7 men and 5 women, median age 49 years) who underwent resection and endoprosthetic reconstruction due to soft tissue sarcoma of the extremity between 2010 and 2021 were analyzed retrospectively. The most common localization was the thigh (66%), and the most common diagnosis myxofibrosarcoma (33%). The most frequent Indications for the endoprosthetic reconstruction after soft tissue tumor resections were the close relationship of the tumor to the bone (*n* = 6), and suspicious bone infiltration on magnetic resonance imaging (MRI) (*n* = 5).

**Results:**

Eight patients (66%) had no evidence of disease at the last follow-up examination (median 62 months), while 4 patients died after an average of 14 months. In 4 of 5 cases in which suspicious bone infiltration was detected on magnetic resonance imaging, the bone lesion was confirmed histopathologically. A Whoops procedure history was significantly negative prognostic in terms of limb survival (*p* < 0.045).

**Conclusions:**

In bone-infiltrating or highly bone surrounding soft tissue sarcomas, wide resection including resection of the affected bone followed by endoprosthetic reconstruction seem to be a recommendable limb-salvage option with good oncological results and acceptable complication rate. The presence of bone infiltration at time of surgery does not increase the risk of local recurrence. A Whoops procedure history significantly reduce the limb survival.

## Introduction

The principle treatment for soft tissue sarcomas (STS) located in the extremities is a complete tumor resection and limb salvage surgery is almost possible [1]. In cases where a wide resection leads to loss of the limb or massive functional deficits, closer but negative margins can be accepted together with (neo-) adjuvant radiotherapy [2]. Moreover, circumferential encasement or direct invasion of bone by an STS often requires additional osseous resection to allow complete removal of the tumor. In these cases, endoprosthetic reconstruction (EPR) of the bone defect may provide satisfactory results as a limb salvage strategy [3].

However, compared to sarcomas of bone origin, STSs requiring EPR after resection present unique challenges. Some of these include advanced patient age, often the need for radiation therapy, decreased chemosensitivity of lesions, and potentially larger defects resulting from muscle compartment resections [2]. In particular, tumors that have invaded bone are larger, relatively deeper-seated, and often metastatic at the time of diagnosis [4]. It is also a known that bone invasion is given relatively little attention in the preoperative evaluation of soft tissue sarcomas [3].

In fact of the rareness of patients with a STS and the need for additionally bone resection we wanted to analyze and present the data of a small cohort from our tertiary sarcoma center. Patients who were diagnosed with soft tissue sarcoma and underwent endoprosthetic reconstruction were reported retrospectively. The aim was to determine indications for bone resection in patients with a STS, surgical and oncological outcomes in these patients and to analyze the factors affecting the outcomes.

## Materials and methods

In this study, 12 patients (male: *n* = 7, female, *n* = 5; median age 49 years (range 23–87 years)) who underwent resection and endoprosthetic reconstruction due to soft tissue sarcoma in a tertiary sarcoma center between 2010 and 2021 were analyzed retrospectively. Data was collected from patient’s records in the medical documentation system.

The diagnoses were as follows: myxofibrosarcoma (*n* = 4), synovial sarcoma (*n* = 3), liposarcoma (*n* = 2), malignant peripheral nerve sheath tumor (*n* = 1), extraskeletalmyxoid chondrosarcoma (*n* = 1) and rhabdomyosarcoma (*n* = 1). The STS were localized as mentioned subsequent: thigh (*n* = 7), cruris (*n* = 1), shoulder (*n* = 1), knee (intraarticularly) (*n* = 1), proximal radioulnar joint (*n* = 1) and both thigh and cruris location (*n* = 1). Exact tumor localizations, tumor grades, presence of bone involvement in imaging and histological evaluation are given in Table 1.

**Table 1:** Patients’ characteristics

*n*	Age (at diagnosis, years)	Localization	Diagnosis, grading	Neoadjuvanttherapy	WhoopProcedureHistory	Recurrencebeforeresection	Suspicious bone infiltration on MRI	boneinfiltration in histology
1	87	cruris, prox.	MFS, G3	-	No	No	yes	yes
2	56	thigh, prox.	MFS, G3	-	Yes	Yes	yes	yes
3	35	thigh, distal	MPSNT, G3	CTx/ILP	No	No	yes	yes
4	50	shoulder	MFS, G1	-	No	No	no	no
5	28	knee, intra-articular	SS	CTx	No	No	no	no
6	51	thigh, distal, cruris prox.	MFS, G3	-	Yes	Yes	no	no
7	23	Prox. radioulnar j	SS, G3	CTx/ILP	Yes	No	no	no
8	59	thigh, prox.	LPS	RTx	No	No	yes	yes
9	52	thigh, distal	EMC	-	No	No	no	no
10	73	thigh, prox.	RMS, G2	CTx	No	No	no	no
11	23	thigh, prox.	SS, G3	CTx	No	No	yes	no
12	54	thigh, distal	LPS, G3	CTx/ILP	No	No	no	no

MPSNT: Malignantperipheral nerve sheathtumor, EMC: extraskeletalmyxoidchondrosarcoma, MFS: myxofibrosarcoma, LPS: Liposarcoma, dedif: dedifferentiatet, RMS: rhabdomyosarcoma, SS: Synovialsarcoma, CTx: chemotherapy, ILP: IsolatedlimbperfusionRTx: radiotherapy, prox: proximal, G: grade, Rx: incompletetumorresection, J: joint

A total of three patients presented with distant metastases at diagnosis. One patient (case 1) hat lung, lymph node and skin metastasis. Another patient (case 3) showed simultaneous bone metastasis. And another patient (case 8) had initially lung metastasis (Table 1).

Most of the patients received neoadjuvant and/or adjuvant treatment. A patient who could not receive chemotherapy due to heart failure received only radiotherapy (RTx) before surgical treatment. Three patients received only chemotherapy (CTx) before treatment (one patient refused treatment after the first cycle due to intolerance). Chemotherapy and ILP (Isolated Limb Perfusion) were applied to three patients before surgical treatment. Four patients received adjuvant RTx, one patient received adjuvant CTx, and three patients received adjuvant CTx and RTx. The different treatment strategies are presented in detail in Table 2. Picci scoring was used to evaluate the tumor histopathological response to neoadjuvant chemotherapy [5].

**Table 2:** Treatment and follow-up

*n*	resection	recon- struction	Response toNeoadj. Therapy	Adjuvanttherapy	Complication	Met at diagnosis	Met at last FU	Amputation	FU (Months)	oncological Status at last FU
1	R0	Tibia intercalary		CTx, RTx	relaps	yes	yes	no	10	DOD
2	R0	Femur intercalary		RTx, CTx, ILP	periprostheticinfection, relaps	no	yes	yes	16	DOD
3	R0	Distal femur	fair	CTx	-	yes	yes	no	21	NED-LTF
4	R0	Prox. humerus		RTx	RTx-associatedfingernecrosis	no	no	no	148	NED
5	R0	Distal femur	total	-	woundhealingdisorder	no	no	no	44	NED
6	R0	Distal femur		-	foot dorsi palsy, periprostheticfr.	no	no	yes	163	NED
7	R0	Prox. ulna	good	RTx	-	no	yes	no	45	NED
8	R0	Prox. femur	fair	-	-	yes	yes	no	11	DOD
9	R0	Distal femur		-	-	no	no	no	31	NED
10	R0	Prox. femur	poor	CTx, RTx	-	no	yes	no	17	DOD
11	R0	Prox. femur	poor	RTx	woundhealingdisorder	no	no	no	22	NED
12	R1	Prox. femur	fair	RTx	-	no	no	no	18	NED- LTF

NED: No evidence of Disease DOD: Dead of disease LTF: Lost to Follow-up, fr: fracture, met. Metastasis, CTx: chemotherapy, ILP: Isolated limb perfusion RTx: radiotherapy, FU: follow- up

**Fig. 1:**
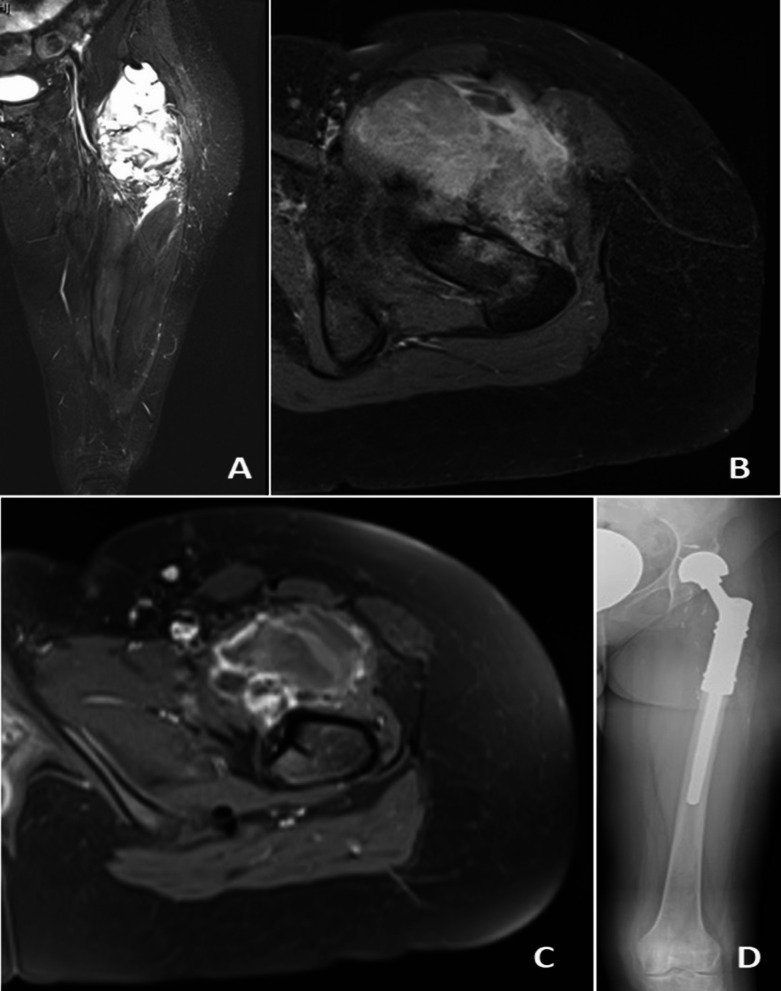
Synovial sarcoma in the thigh (case no 11), (**a**) coronal non-contrast T2-weighted MRI section, (**b**) a signal change in the medullary space in the axial non-contrast T2-weighted MRI (**c**) no bone lesion was detected in the post-contrast axial T1-weighted fat-suppressedMRI after neoadjuvant treatment, (**d**) X- ray (a.p.) of the left femur with resection of the proximal femur and reconstruction with a tumor prosthesis.No bone invasion was found in the histopathological examination of the final resection

**Fig. 2:**
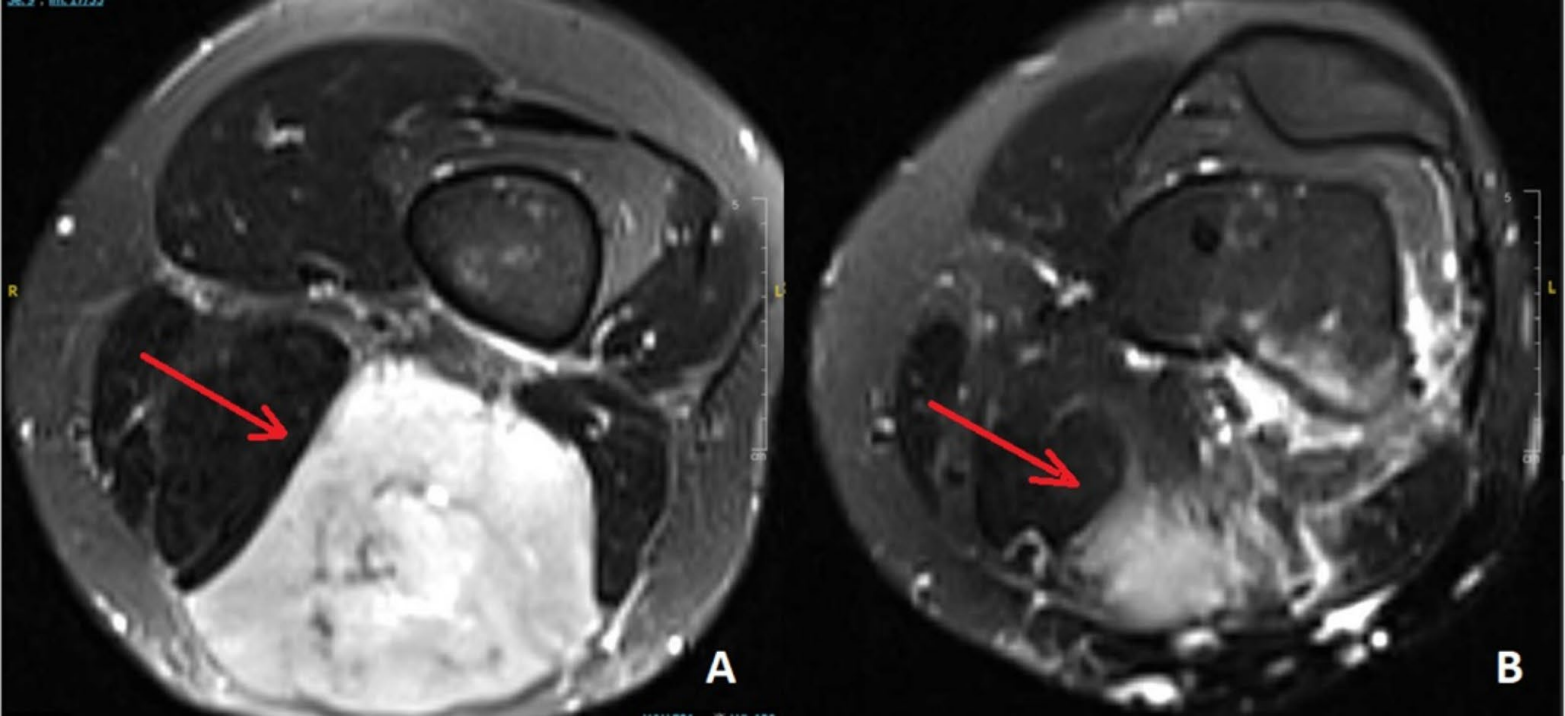
MPNST in the distal thigh (case 3), (**a**) soft tissue mass in the non-contrast T2 axial MRI section. (**b**) Medullary signal changes in the femur are seen in the more distal part of the mass. Bone metastasiswas found in the histopathological examination of the final resection. Red arrow shows soft tissue mass

**Fig. 3:**
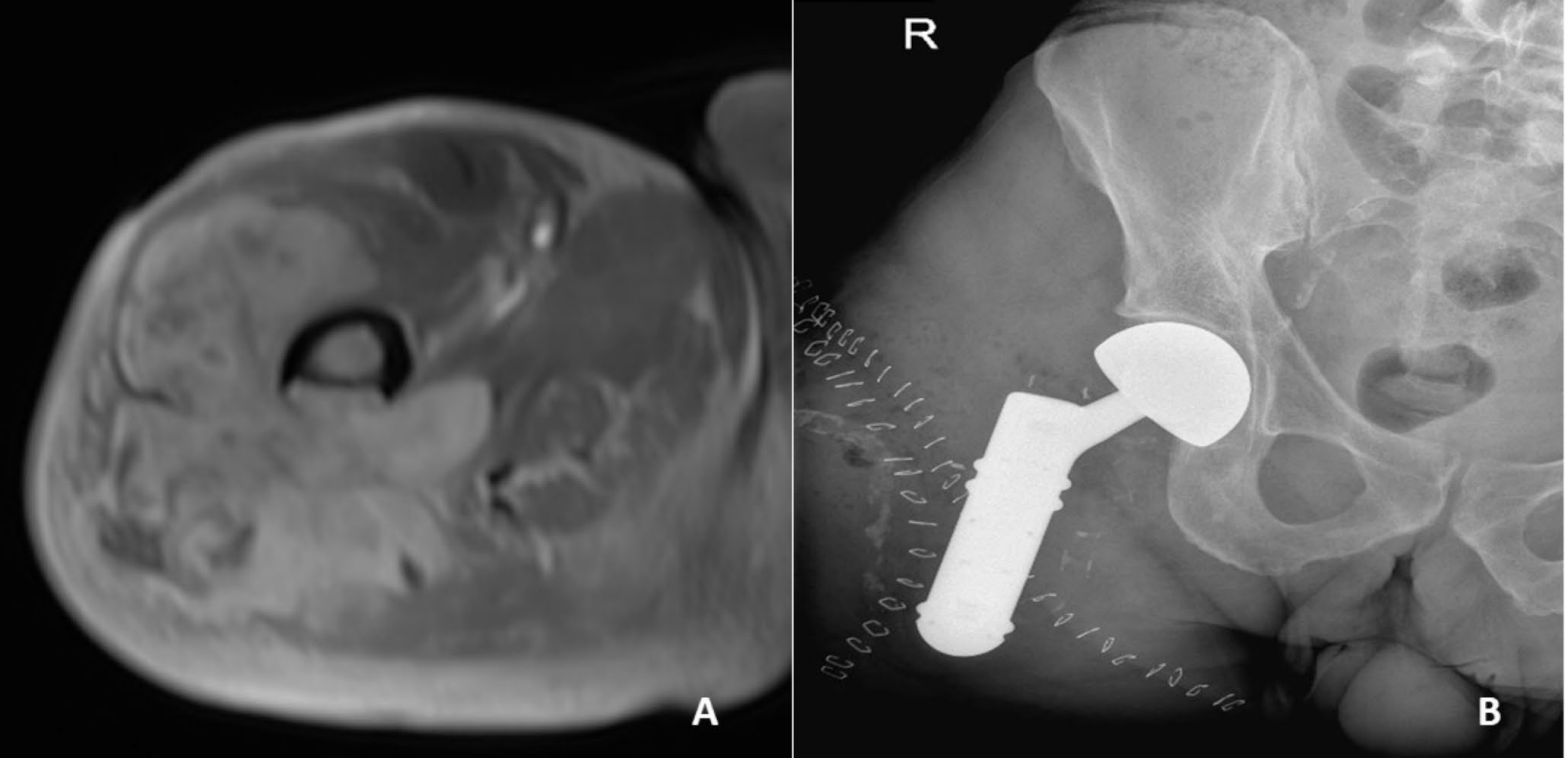
(**a**) Dedifferentiated liposarcoma in the right proximal thigh, bone invasion (cortical erosion) visible in axial post-contrast MRI image (Patient no 8) (**b**) hip joint disarticulation followed by stump reconstruction with a proximal femur tumorprosthesis; bone invasion was found in the histopathological examination of the final resection

**Fig. 4:**
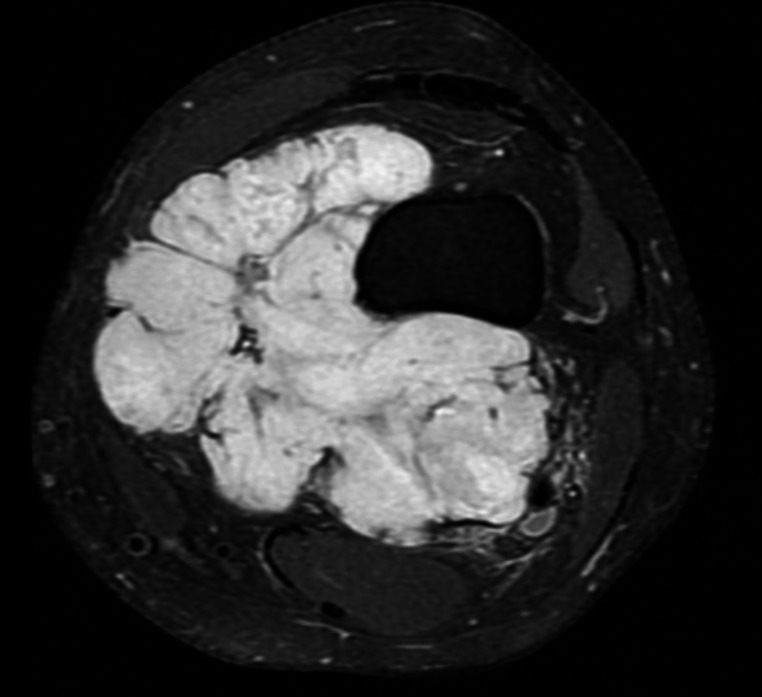
Extraskeletalmyxoid chondrosarcoma in the area of the popliteal fossa and the medial knee compartment in the axial non-contrast Proton Density Fat Saturation MRI image (Patient no 9). The bone was surrounded by more than 180 degrees. No bone invasion was found in the histopathological examination of the final resection

Indications for supplementary bone resection in this patient cohort were the close relationship of the tumor to the bone (*n* = 6), suspicious bone infiltration on MRI (*n* = 5), and the presence of an intra-articular tumor with a history of prior arthroscopic biopsy (*n* = 1). Evidence of cortical erosion or signal change in the bone marrow space on MRI was defined as “suspicious bone infiltration on MRI”(Figs. 1, 2 and 3). “Close relationship of the tumor to the bone” was defined as: MRI showed that the lesion was adjacent to the bone, and the patient preoperatively and intraoperatively was examined whether the sarcoma moved freely on the periosteal surface of the bone, andin this examination, the tumor appeared fixed to bone, or (2) on MRI, the tumor surrounds the bone 360 ​​degrees or close to 360 degrees, to the extent that R0 resection cannot be achieved without bone resection(Fig. 4).

Three patients who previously underwent Whoops procedure (unplanned sarcoma resection) in other centers were included in this study. In these cases, preoperative diagnoses were noted as hematoma (case 2), benign soft tissue swelling (case 6), and ganglion (case 7). In one patient (case 5), arthroscopic biopsy was performed for an intra-articular mass in the knee with the preliminary diagnosis of ganglion.

Whenour surgeries are examined; wide resectionwas performed in 11 patients, and R1 resectionwas obtained in one patient (who refused a secondary amputation to gain a complete resection). Extraarticular resection was necessary in a patient with an intra-articular tumor and in a patient with a large tumor located in the distal thigh and the proximal cruris (case 6).

Excluding one patient who underwent hip disarticulation, the average resection length was 14.9 cm (range 6–35 cm) in a total of 11 patients. The average resection length was 16.4 (range 8–35) in the lower extremity and 8.2 (range 6-10.5) in the upper extremity. Defect reconstruction was performed using the Modular Universal Tumor and Revision System (MUTARS^®^; Implantcast, Buxtehude, Germany) for endoprosthetic reconstruction. The most frequently used prostheses for the reconstruction were a proximal femur tumorendoprosthesis and a distal femur tumorendoprosthesis (four cases each). All applied reconstructions are given in Table 2. In the patient in whom a femoral intercalary prosthesis was used, a Buxtehuder stem was applied due to the short remaining proximal femur bone stock after resection [6]. The patient, who underwent hip disarticulation, underwent stump reconstruction with femoral proximal tumor endoprosthesis (Fig. 3).

### Statistical analysis

Statistical analysis was performed by IBM SPSS version 22.0 (IBM Corp., Armonk, NY, USA) software. Categorical variables were given as numbers and percentages. In 2 × 2 comparisons between categorical variables, Pearson Chi-square test was used if the expected value was (> 5), Chi-square Yates test was used if the expected value was between (3–5), and Fisher’s Exact test was used if the expected value was (< 3). Kaplan Meier analysis was performed for metastasis-free survival. P- value < 0.05 was considered statistically significant.

## Results

### Oncological results

Eight patients (66%) had no evidence of disease at the mean 62 months follow-up (range 18–163), while 4 patients died after an average of 14 months (range 10–17).

Of the seven patients who received a neoadjuvant treatment, one patient showed histologically total, one patient good, and two patients fair response while the other three patients showed a poor response histologically in the resection specimen (Table 2).

**Fig. 5:**
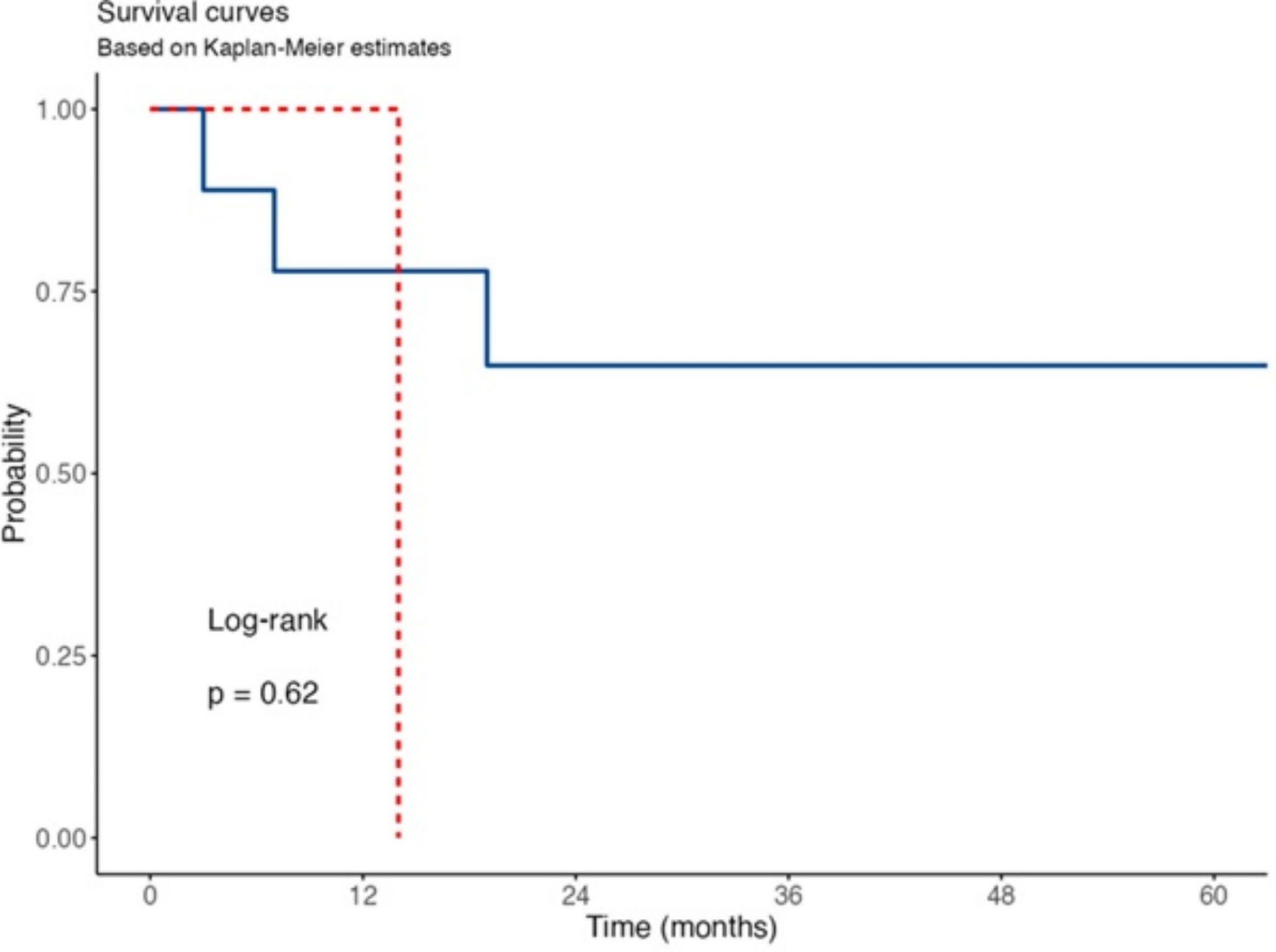
Kaplan Meier analysis for metastasis free survival

Six of the 12 patientsinitially had localized disease and remained disease-free without developing recurrence or metastasis during follow-up. Two patients are under disease-free follow-up after metastasectomy. While two cases initially had localized disease, metastasis developed in follow-up and they died due to metastasis progression. Two patients, who were metastatic at the time of diagnosis, died due to metastasis progression. For detailed information about the cases, see Tables 1 and 2. Kaplan Meier analysis for metastasis-free survival is given in Fig. 5.

In 4 of 5 cases in which suspicious bone infiltration was detected on magnetic resonance imaging, the bone lesion was confirmed histopathologically (case 1,2,3, and 8). However, no tumor was found in the histopathological examination in one case with medullary signal changes and in which the bone was resected due to suspicion of tumor (case 11).

The correlation between death and the precence of a Whoops Procedure historywas analyzed. While one (33%) of the three patients who had history of R1 resectiondied during the follow-up period, three (33%) of nine patients with no previous R1 surgery, died.

The fact that the tumor was located in the thigh, the presence of metastases at the time of diagnosis, a history of a Whoops Procedure, the diagnosis of a myxofibrosarcoma, the presence of bone infiltration, CTx and ILP treatment, and biopsy in a sarcoma center did not significantly affect the recurrence and survival of the patient (respectively, *p* = 1.000, *p* = 0.455, *p* = 0.455, *p* = 0.091, *p* = 0.152, *p* = 1.000, *p* = 0.515, *p* = 1.000, *p* = 0.236, *p* = 1.000, *p* = 0.547, *p* = 0.067, *p* = 0.491, *p* = 1.000).

### Complications, revision surgeries and limb survival

Postoperative wound healing problems occurred in two patients (17%). While a patient with superficial skin necrosis was treated without surgery (case 11), the other patient with wound dehiscence and fatty tissue necrosis (most likely with suture intolerance) underwent debridement and secondary wound closure (case 5).

Periprosthetic infection occurred in one patient (8%) three months after surgery (case 2). The patient received a cement spacer after prosthetic explantation. Amputation followed in this multimetastatic patient due to aggressive recurrence in the 4th month postoperatively. Complications are summarized in Table 2.

In one patient (8%), who underwent extra-articular resection due to tumor in both the distal thigh and proximal cruris, implant revision was performed due to wear in the joint mechanism after 65 months (case 6). Amputation was performed seven years later in this patient because of periprosthetic fracture and aseptic loosening.

Overall, two of the twelve patients (17%) finally had to undergo amputation, on average 78 months after prosthesis implantation (4–151 months).

A history of Whoops Procedurewas a significantly negative prognostic factor in terms of limb survival (*p* < 0.045). The tumor localization in the thigh, the presence of metastases at the time of diagnosis, the diagnosis of a myxofibrosarcoma, the presence of bone infiltration, CTx and ILP treatment and biopsy in a sarcoma center did not significantly affect limb survival (respectively, *p* = 0.515, *p* = 1.000, *p* = 0.091, *p* = 1.000, *p* = 1.000andp = 0.515).

## Discussion

The current study aimed to analyze the indications as well as oncological results and complications of tumor resection and reconstruction with tumorendoprostheses in soft tissue sarcomas ambient to the adjacent bone. The most striking finding of the presented study was that the common indication for bone resection was not a radiologically detected bone invasion, but the presence of “a close relationship of the tumor to the bone” in the preoperative radiological evaluation with 50% of the presented cases. Another important finding was that the presence of a history ofWhoops procedurehad a statistically significant negative impact on limb survival. Third, the presence of histologicallaybone invasion did not increase the risk of recurrence after resection and EPR. On the other hand, another important result of this study was the existence of high limb salvagerates (83%) in this special patient cohort.

Studies on bone resections and reconstructions with endoprostheses in soft tissue sarcomas are quite limited in the literature and case series generally report data on an isolated body location or specific tumor group [1–4, 7, 8]. But in the current study, bone resections and endoprosthetic reconstructions was performed for a variability of localizations and soft tissue sarcoma entities. The most common indication was the close relationship of the tumor to the bone, followed by the presence of suspicious bone infiltration on magnetic resonance imaging. The presence of bone invasion in preoperative MRI seems to show a clear indication for resection of the affected bone. Even if there is no bone invasion in radiological examinations, the presence of a mass that covers almost the entire bone or surrounds it 360 degrees, additionally bone resection should be performed to achieve a complete tumor resection. In case of no bone invasion and no signal change in the bone marrow space, but the lesion adjacent to bone in preoperative MRI, tumor mobility should be examined manually preoperatively and intraoperatively. Examination is performed to determine whether the sarcoma moves freely on the periosteum of the bone or whether the tumor is fixed to the bone. If the sarcoma “rolls” onto the bone, a subperiosteal resection is performed. Even on intraoperative examination after opening the fascia, if the sarcoma appears fixed to the bone, the bone should be resected [1–4, 7, 9].

Interpretation of preoperative MRI findings should be made with extreme caution. In this study, bone was resected in the presence of evidence of cortical erosion on preoperative MRI (cases 1, 2, and 8). And tumor infiltration was confirmed histopathologically in all of them. In the presence of medullary signal change, it is more challenging to differentiate between tumor and edema. While no tumor was found in the histopathological examination in one of the two cases with medullary signal changes and in which the bone was resected due to suspicion of tumor (cases 11) (Fig. 1), the tumor was confirmed in one (case 3)(Fig. 2). In the case with no histopathological confirmation of tumor, the medullary signal can be an edema. On the other side, histopathological tumor confirmation may not have been possible in this casebecause the patients with medullary signal change received neoadjuvant treatment before surgery. In Fig. 1, it can be seen that the existing medullary change disappeared after neoadjuvant treatment and the size of the soft tissue mass decreased.

Unplanned and often incomplete excisions of sarcomas due to suspected benign diagnoses continue to be performed frequently and called as Whoops procedure. These interventions negatively affect the type and extent of definitive surgery and require advanced soft tissue reconstructive procedures more frequently. These cases are associated with a higher number of reoperations and a higher risk of infection, and the risk of amputation is increased in these cases [10, 11]. In the presented study, there was a history of whoops procedure in three cases. In these cases we performed complete tumor resection along with bone resection but limb survival was significantly reduced. Similarly in the study of Traub et al., the amputation rate was significantly increased in the group with unplanned surgery compared to the planned surgery group [10].

Lin et al. examined nonmetastatic, high-grade soft tissue sarcomas abutting the bone that have a tumor size > 5 cm and which were located in the lower extremities. In the case series with 50 patients, three cases had a histopathologically proven bone invasion (6%). The authors found no significant difference in terms of recurrence between cases in which bone resection was performed and cases in which only soft tissue resection was performed [7]. The results are in line with the current study. It was shown that the presence of bone invasion did not increase the risk of recurrence. In another study, Panicek et al. also reported that the presence of bone invasion on preoperative MRI did not increase the risk of recurrence after complete resection [12].

Rowel et al. reported a series of 29 STS cases of the lower extremities with radiologically bone invasion in which EPR was applied [2]. They compared the data with the STS group without an indication for EPR and found that survival was reduced in these cases. Nine patients had deep infection surgeries and two received subsequent amputations in the bone invasion group. The authors reported that the prosthesis survival was lower in this group. Yan et al. analyzed a cohort of 30 STS patients with juxtaarticular bone invasion in which they applied EPR [3]. They achieved satisfactory results with acceptable complication rates (17%). Nakamura et al. reported high complication rates (infection, *n* = 9, aseptic loosening, *n* = 4, local recurrence, *n* = 9) in a series of 27 high-grade STS in a multicenter study on EPRs in lower extremity STS [4]. Ferguson et al. reviewed 48 cases and found out that histological bone invasion was a poor prognostic factor for overall survival [1]. In the current study, which included all indications for bone resection in soft tissue sarcomas (intraarticular tumor mass, histopathological bone infiltration and radiological bone-association), 83% limb survival was achieved. Therefore we think that this treatment pathway offers a satisfactory option for these selected patients with regard to oncological and functional results.

The histological subtype of soft tissue sarcoma can provide information about its behavior. While subperiosteal resection is usually sufficient for a well-differentiated liposarcoma, high-grade tumors such as synovial sarcoma, undifferentiated pleomorphic sarcoma, and malignant peripheral nerve sheath tumor generally tend to erode the bone and may require resection of the affected segment [3, 13]. In studies where bone resection and endoprosthestic reconstruction were performed due to soft tissue sarcoma, the most common diagnosis was generally undifferentiated pleomorphic sarcoma (previously known as malignant fibrous histiocytoma), followed by liposarcoma, synovial sarcoma, leimyosarcoma or myxofibrosarcoma in different studies [1, 3, 4]. Considering that the relationship of the soft tissue mass with bone is often underestimated in the preoperative evaluation, it can be emphasized that attention should be paid to bone invasion in especially these histologically subtypes. However, in this current study, the most common tumor location was the thigh, and this data was consistent with the literature [1, 2, 4].

This study had some limitations. The study was primarily retrospective and included the results of a single, but well experienced sarcoma center. Although every living patient was followed for at least 12 months, follow-up periods – especially regardinglimb survival - were relatively short. The number of cases was limited due to the fact thatsoft tissue sarcomas are scarce and bone resection is rarely needed in these cases. The small case number limited statistical power. However, these data come from a well-equipped and specialized center with experienced surgeons and the study includes data over a wide period. There are valuable in terms of better understanding this rare group of cases and hopefully gives other surgeons some assistance to handle these special cases.

## Conclusion

In conclusion, complete STS resection including a resection of the associated bone segment followed by endoprosthetic reconstruction seem to be a recommendable option in selected cases. The most common indication undergoing bone resection is close relationship of the tumor to the bone in patients with STS. The presence of bone infiltration does not increase the risk of local recurrence. Unplanned intralesional resection (Whoops procedure) significantly reduce the limb survival. Future prospective multicenter studies with larger patient numbers are needed to better understand the indications and outcomes of endoprosthetic replacement in bone-associated soft tissue sarcomas.

## Data Availability

The data that support the findings of this study are available on request from the corresponding author.
